# Necrotising retinopathy-like lesions as a manifestation of ocular sarcoidosis

**DOI:** 10.1186/s12348-018-0154-7

**Published:** 2018-08-03

**Authors:** Florence Rasquin, Nacima Kisma, Laure Van Bol

**Affiliations:** Department of Ophthalmology, Erasme Hospital, Université Libre de Bruxelles, route de Lennik 808, 1070 Brussels, Belgium

**Keywords:** Necrotising, Retinopathy, Sarcoidosis, Lymphoproliferative, Virus

## Abstract

**Background:**

A 56-year-old Caucasian man presented with a 2-weeks history of decreased vision in the right eye. Vitritis, papillitis, cystoid macular oedema and inferior diffuse retinal infiltration were noticed. Extensive blood work-up, anterior chamber paracentesis with polymerase chain reaction (PCR) and Goldmann-Witmer coefficient, tuberculin skin test (PPD-test), fluorodeoxyglucose Positron Emission Tomography CT scan (FDG-PET/CT), lymph node biopsy and pars plana vitrectomy were performed.

**Results:**

Aqueous and vitreous samples were negative for an infectious and a lymphoproliferative etiology. Enlarged hilar and mediastinal lymph nodes were detected by FDG-PET/CT and subsequently biopsied, allowing to confirm the diagnosis of sarcoidosis. After a few months of oral corticosteroid therapy, the inflammation resolved completely and was replaced by atrophic retinal scars.

**Conclusion:**

Necrotising retinopathy-like lesions mimicking an infectious process or a lymphoproliferative disorder can be an atypical manifestation of ocular sarcoidosis.

## Introduction

Sarcoidosis is a chronic systemic disease of unknown origin. The disorder affects the eyes with a reported incidence of 30–60% during the course of the disease [1].

Recently, four levels of certainty for the diagnosis of ocular sarcoidosis were defined from various diagnostic criteria including suggestive clinical signs, investigational procedures and biopsy results. However, the histopathological confirmation remains the gold standard for the definitive diagnosis of sarcoidosis [1].

Ocular sarcoidosis may present with a broad variety of ocular features. In this study, we report the case of necrotising retinopathy-like lesions, highlighting yet again the widespread spectrum of ocular features that can be observed in the disease.

## Findings

A 56-year-old Caucasian man presented with a 2-week history of decreased vision in the right eye. He also related experiencing a dry cough for 2 months. Noticeably, he reported that he had allegedly been diagnosed with pulmonary sarcoidosis 30 years earlier following a CT scan and a blood workup. However, no biopsy was performed at the time to confirm the diagnosis as the patient had refused to undergo a mediastinoscopy. He did not report any previous episode of uveitis.

**Fig. 1:**
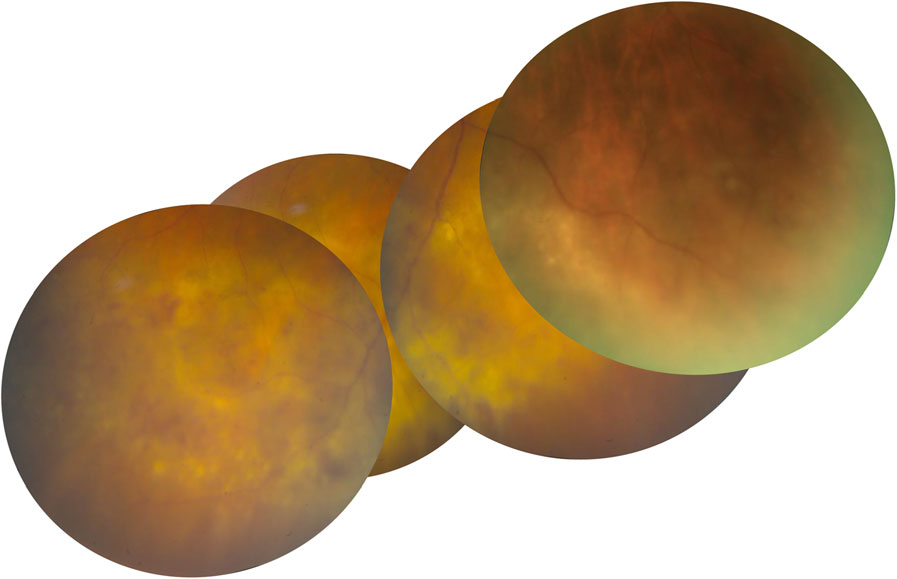
Fundus photograph of the inferior retina of the right eye showing a white diffuse retinal infiltration associated with haemorrhages

**Fig. 2:**
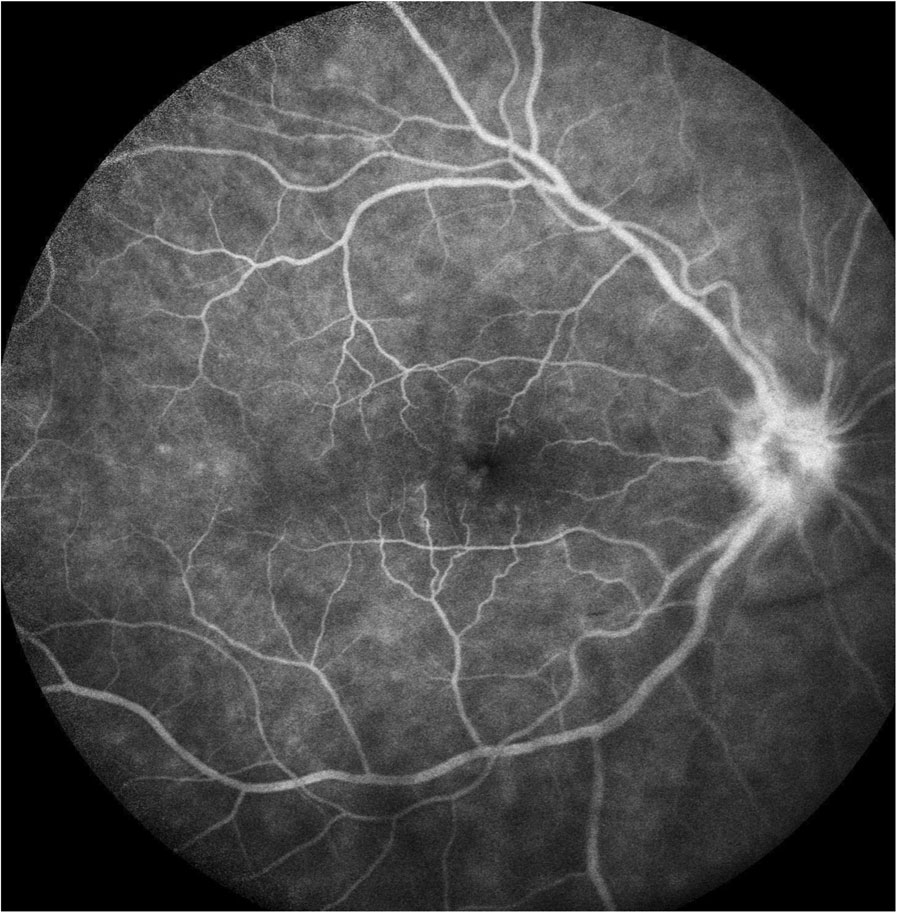
FA of the right eye displaying papillitis and a mild cystoid macular edema

**Fig. 3:**
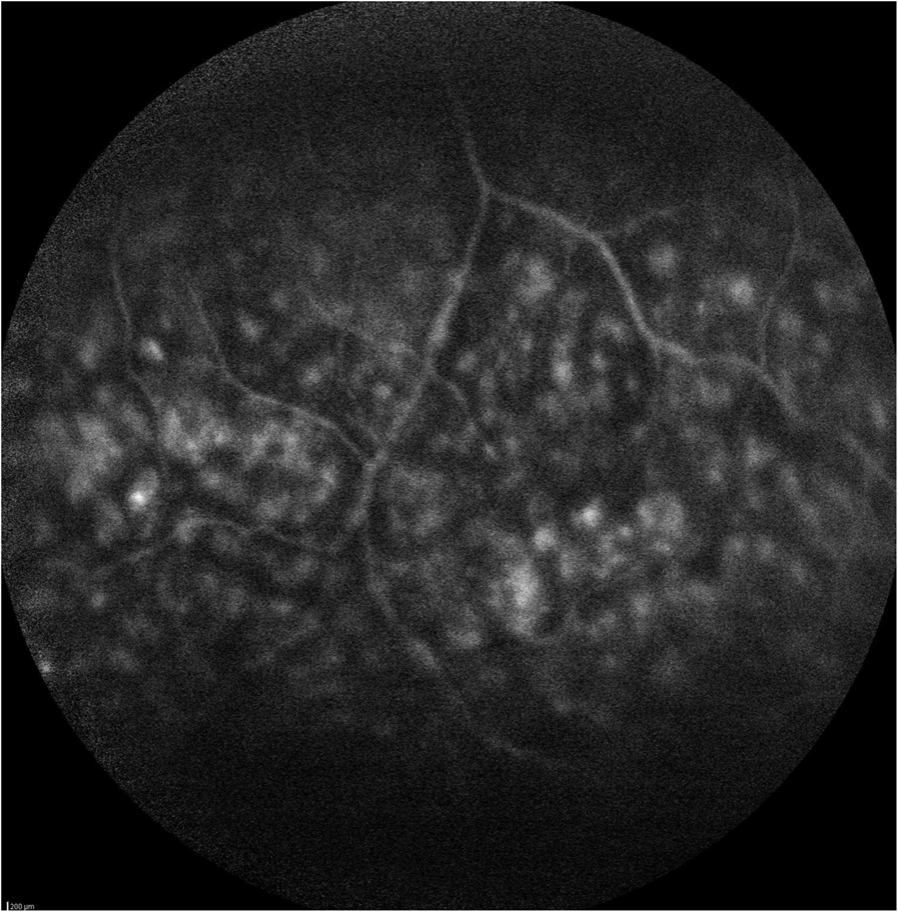
FA demonstrating a hyperfluorescent mottling of the lesion and capillary leakage

**Fig. 4:**
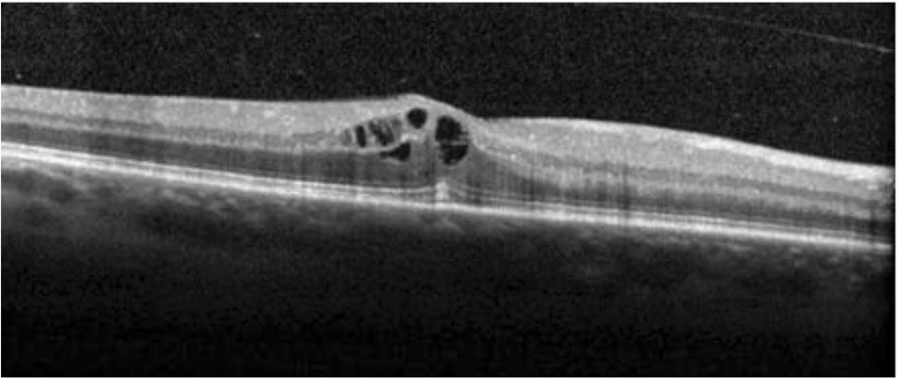
SD-OCT showing intraretinal fluid in the foveal area

On examination, his best-corrected visual acuity was 0.5 in the right eye and 1.0 in the left eye. Slit lamp examination revealed quiet anterior chambers in both eyes, but the patient was using steroid drops prescribed by his general ophthalmologist. Vitritis evaluated at 1+ was present in his right eye. Intraocular pressure was normal in both eyes. Fundus examination of the right eye revealed the presence of a white diffuse infiltrate located in the inferior retina associated with round hemorrhages (Fig. 1). The left fundus examination was unremarkable. Fluorescein angiography (FA) of the right eye showed papillitis, cystoid macular oedema confirmed by spectral domain optical coherence tomography (SD-OCT) and a mottled hyperfluorescence in the area of the retinal lesion (Figs. 2, 3, and 4). Indocyanin green images provided no additional information.

An anterior chamber paracentesis with evaluation of the aqueous humour by polymerase chain reaction for Herpes Simplex virus 1 and 2, Epstein-Barr virus, cytomegalovirus, toxoplasmosis and Goldmann-Witmer coefficient for toxoplasmosis was performed. Serologic testing for HIV, syphilis, herpes viruses, and toxoplasmosis as well as the serum angiotensin converting enzyme and lysozyme levels were also ordered.

## Results

The PCR analysis and the Goldmann-Witmer coefficient were negative for a viral etiology and toxoplasmosis. The serologic screening and the PPD-test were also negative. The angiotensin converting enzyme level was normal at 25 U/L (normal range 8–55 U/L), but there was a slight increase in the serum lysozyme level to 19.1 μg/ml (normal range < 17 μg/ml). The FDG-PET/CT showed an intense FDG uptake in the hilar and mediastinal lymph nodes. A biopsy was subsequently performed and revealed the presence of non-caseating granulomas, confirming the diagnosis of sarcoidosis. The patient was started on oral steroids (methylprednisolone 64 mg per day). Two weeks later, the vision in his right eye improved to 1.0, and we noticed a significant improvement in vitritis associated with the disappearance of the cystoid macular oedema on SD-OCT. Following treatment initiation, the retinal infiltrates appeared initially more granulomatous with a slight increase in the number of retinal haemorrhages. Over time, the area of retinal necrosis appeared more and more circumscribed with a resolution of the haemorrhages and was gradually replaced by scarring lesions. The steroid treatment was gradually tapered. Unfortunately, 2 months later, as the patient was under 12 mg per day of methylprednisolone, the vitritis and the macular oedema recurred with a drop in vision to 0.7. After a transient interruption of his steroid treatment for 1 month, vitrectomy was therefore performed in order to exclude a primary intraocular lymphoma. The vitreous sample showed some macrophages and a few small and mature lymphocytes. Cytologic evaluation did not highlight any signs of malignancy. Lymphocyte typing could not be performed because the number of lymphocytes was insufficient. Cerebral magnetic resonance imaging showed no specific lymphomatous lesion.

**Fig. 5:**
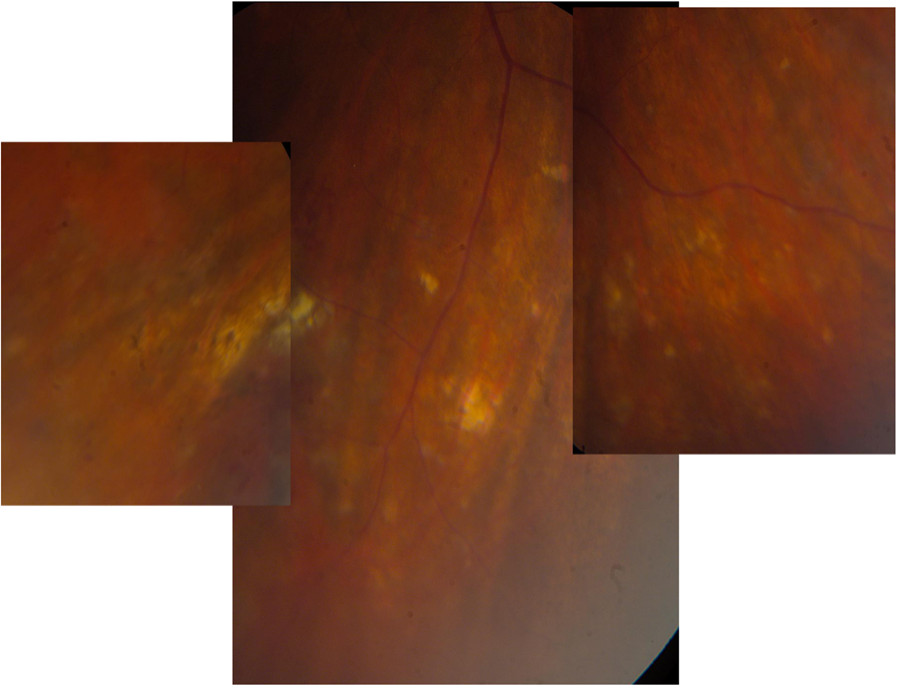
Fundus photograph 6 months after the withdrawal of steroids showing thin atrophic scars and the disappearance of the white diffuse infiltration

The dose of oral steroid was increased again and then gradually tapered over a period of 9 months. Six months after the withdrawal of steroids, there were no signs of recurrence. The vision of the right eye was 1.0. Dilated fundoscopy showed that thin atrophic scars had replaced the white diffuse infiltration on the inferior retina (Fig. 5). No pigment alterations were observed.

## Discussion

Sarcoidosis is an inflammatory disease of unknown origin characterized by non-caseating granulomas. Various organs may be affected including the lung, lymph nodes, skin, heart, liver, muscles, and the eyes. Ocular sarcoidosis can present with a myriad of signs and symptoms including anterior, intermediate and/or posterior uveitis. Posterior segment lesions are reported to occur in approximately 14–28% of patients with ocular sarcoidosis. The main findings are vitritis, periphlebitis, chorioretinitis, choroidal nodules, and retinal neovascularization [2]. In our patient, the diagnosis of ocular sarcoidosis was based on the diagnostic criteria published by the International Workshop on Ocular Sarcoidosis (IWOS) in 2009 [1], and the biopsy of mediastinal lymph nodes provided the histological confirmation.

To our knowledge, the necrotising retinopathy-like lesions seen in this patient are unusual for ocular sarcoidosis. Nevertheless, a case of necrotising retinopathy simulating acute retinal necrosis and causing rhegmatogenous retinal detachment has already been described in a patient with sarcoidosis [3]. The atypical presentation in our patient explains why sarcoidosis was not the first diagnosis to be suspected, despite his possible history of sarcoidosis. In our patient, the resolution of the lesion differs from the typical fundus appearance observed in the late stages of the acute retinal necrosis syndrome. The white infiltration of the retina disappeared leaving a slightly altered retina without pigment alterations.

Other causes of retinitis that simulate acute retinal necrosis have long been established. Non-viral infectious agents including toxoplasma, syphilis, aspergillus, and other entities such as Behçet disease or intraocular lymphoma has been reported [4].

The recurrence of inflammation that was observed during the steroid taper had suggested the development of an intraocular lymphoma in addition to the underlying diagnosis of sarcoidosis; however, histological examination of the vitreous sample could not confirm this hypothesis. We suppose that the initial tapering of steroids was too fast and therefore led to the recurrence of the inflammation.

In conclusion, ocular sarcoidosis may present with a wide variety of ocular signs. Necrotising-like retinal lesions should also be considered as part of the disease spectrum.

## Abbreviations

FA: Fluorescein angiography
FDG-PET/CT: Fluorodeoxyglucose positron emission tomography CT scan
PCR: Polymerase chain reaction
PPD-test: Purified protein derivative test
SD-OCT: Spectral domain optical coherence tomography
